# Purifying selection decreases the potential for Bangui orthobunyavirus outbreaks in humans

**DOI:** 10.1093/ve/vead018

**Published:** 2023-03-08

**Authors:** Gregory S Orf, Lester J Perez, Todd V Meyer, Ka-Cheung Luk, Kenn Forberg, Mary A Rodgers, Abbas Hadji, Linda James, Samuel Mampunza, Asmeeta Achari, Guixia Yu, Scot Federman, Charles Y Chiu, Carole A McArthur, Gavin A Cloherty, Michael G Berg

**Affiliations:** Abbott Laboratories and Abbott Pandemic Defense Coalition, Abbott Park, IL 60064, USA; Abbott Laboratories and Abbott Pandemic Defense Coalition, Abbott Park, IL 60064, USA; Abbott Laboratories and Abbott Pandemic Defense Coalition, Abbott Park, IL 60064, USA; Abbott Laboratories and Abbott Pandemic Defense Coalition, Abbott Park, IL 60064, USA; Abbott Laboratories and Abbott Pandemic Defense Coalition, Abbott Park, IL 60064, USA; Abbott Laboratories and Abbott Pandemic Defense Coalition, Abbott Park, IL 60064, USA; Abbott Laboratories and Abbott Pandemic Defense Coalition, Abbott Park, IL 60064, USA; Université Protestante au Congo, Kinshasa, Democratic Republic of the Congo; Université Protestante au Congo, Kinshasa, Democratic Republic of the Congo; Department of Laboratory Medicine, University of California San Francisco, San Francisco, CA 94143, USA; Department of Laboratory Medicine, University of California San Francisco, San Francisco, CA 94143, USA; Department of Laboratory Medicine, University of California San Francisco, San Francisco, CA 94143, USA; Department of Laboratory Medicine, University of California San Francisco, San Francisco, CA 94143, USA; Department of Medicine, Division of Infectious Diseases, University of California San Francisco, San Francisco, CA 94143, USA; University of Missouri-Kansas City, Kansas City, MO 64110, USA; Abbott Laboratories and Abbott Pandemic Defense Coalition, Abbott Park, IL 60064, USA; Abbott Laboratories and Abbott Pandemic Defense Coalition, Abbott Park, IL 60064, USA

**Keywords:** Bangui virus, bunyavirus, the Democratic Republic of the Congo, virus discovery, febrile illness

## Abstract

Pathogens carried by insects, such as bunyaviruses, are frequently transmitted into human populations and cause diseases. Knowing which spillover events represent a public health threat remains a challenge. Metagenomic next-generation sequencing (mNGS) can support infectious disease diagnostics by enabling the detection of any pathogen from clinical specimens. mNGS was performed on blood samples to identify potential viral coinfections in human immunodeficiency virus (HIV)-positive individuals from Kinshasa, the Democratic Republic of the Congo (DRC), participating in an HIV diversity cohort study. Time-resolved phylogenetics and molecular assay development assisted in viral characterization. The nearly complete genome of a novel orthobunyavirus related to Nyangole virus, a virus previously identified in neighboring Uganda, was assembled from a hepatitis B virus–positive patient. A quantitative polymerase chain reaction assay was designed and used to screen >2,500 plasma samples from Cameroon, the DRC, and Uganda, failing to identify any additional cases. The recent sequencing of a US Center for Disease Control Arbovirus Reference Collection revealed that this same virus, now named Bangui virus, was first isolated in 1970 from an individual in the Central African Republic. Time-scaled phylogenetic analyses of Bangui with the related Anopheles and Tanga serogroup complexes indicate that this virus emerged nearly 10,000 years ago. Pervasive and episodic models further suggest that this virus is under purifying selection and that only distant common ancestors were subject to positive selection events. This study represents only the second identification of a Bangui virus infection in over 50 years. The presumed rarity of Bangui virus infections in humans can be explained by its constraint to an avian host and insect vector, precluding efficient transmission into the human population. Our results demonstrate that molecular phylogenetic analyses can provide insights into the threat posed by novel or re-emergent viruses identified by mNGS.

## Background

Metagenomic next-generation sequencing (mNGS) of patient specimens enables the detection of any pathogen otherwise undetectable with conventional molecular methods. While its full clinical impact has yet to be realized, there are clear examples demonstrating that mNGS can lead to an actionable diagnosis and treatment (Wilson et al. 2014, 2019; Schlaberg et al. 2017; Greninger 2018; Hogan et al. 2021). Another benefit of this technology has been its utility in discovering many novel viruses over the last two decades (Tang and Chiu 2010; Radford et al. 2012; Chiu 2013). Insect vectors (Li et al. 2015; Shi et al. 2016; Sadeghi et al. 2018; Kapuscinski et al. 2021), rodent and bat hosts (Hu et al. 2017; Oude Munnink et al. 2017; Yinda et al. 2018), and patient cohorts with unexplained illness (Phan et al. 2018; Thi Kha Tu et al. 2020; Berg et al. 2021a) are all fertile ground for virus hunters. Immunocompromised individuals susceptible to microbes not typically tested for, and otherwise readily cleared by healthy people, can be harbingers of novel or re-emergent viruses and thus represent another population in need of monitoring (Zinter et al. 2019; Pruitt 2021). Indeed, serving as incubators for virus evolution, immunocompromised patients with prolonged SARS-CoV-2 infections are believed to have given rise to numerous COVID-19 variants capable of evading immunity (Kemp et al. 2021; Sonnleitner et al. 2022).

Viruses transmitted by insects (arboviruses) are frequent causes of acute febrile illness, encephalitis, rashes, and hemorrhagic fever. Bunyavirus genera including *Nairovirus*, *Orthobunyavirus*, and *Phlebovirus* are arboviruses that infect vertebrates and are vectored by hematophagous flies, mosquitos, culicoid midges, and ticks (Horne and Vanlandingham 2014). The reassortment of their segmented, negative-sense RNA genomes contributes to their genetic diversity and pathogenicity (Briese, Calisher, and Higgs 2013). As natural habitats are destroyed or encroached upon, thus increasing contact with vectors, bunyaviral infections in humans (e.g. severe fever with thrombocytopenia syndrome, La Crosse encephalitis, Crimean–Congo hemorrhagic fever, and Rift Valley fever) and livestock (e.g. Schmallenberg, Nairobi sheep, and Ngari) have been on the rise (Braack et al. 2018; Rodriguez et al. 2020; Mayanja et al. 2021). Not surprisingly, as the adoption of mNGS methods has gained traction, increasing numbers of novel, disease-causing bunyaviruses have recently been described (e.g. see Simo Tchetgna et al. 2019; Rodriguez et al. 2020).

The *Orthobunyavirus* genus (*Peribunyaviridae*) consists of >220 species classified into 18 serogroups based on serological relatedness, as determined by complement fixation, neutralization, and hemagglutination inhibition (Elliott 2014). Many are found in the Amazon region and isolated from mosquitos or non-human hosts although several can cause diseases in humans (Dias et al. 2022). Some of these orthobunyaviruses cause large (e.g. Oropouche; Vasconcelos et al. 2011; Files et al. 2022) and small (e.g. La Crosse; Bennett et al. 2007) outbreaks that can result in greater diversification, whereas other species in the genus consist of relatively few reported strains, suggesting that infections caused by these species result from sporadic spillover events (Grubaugh et al. 2019). The ability to discern a priori whether a novel virus represents an epidemic or pandemic threat could inform timely and appropriately targeted public health responses. Here, we report the discovery and phylogenetic characterization of a second strain of Bangui orthobunyavirus that demonstrates the potential role of time-resolved phylogenetics in making disease threat predictions.

## Methods

### Patients and sample collection

Specimens were collected as part of a human immunodeficiency virus (HIV) viral diversity study in 2017–9 as previously described (Pour et al. 2020; Berg et al. 2021b). The investigation protocol conformed to local regulations and was approved by the Université Protestante au Congo ethics committee in Kinshasa; ethics approval was received by memorandum dated May 2017 via #CEUPC-0027. Informed verbal consent in the Democratic Republic of the Congo (DRC) was obtained from all participants in either French or Lingala, and plasma samples were de-identified for analysis. The Institutional Review Board (IRB) approval from the University of Missouri-Kansas City Research Board for protocol 16-411 was approved on 18 October 2016. The IRB approval for metagenomic sequence analysis of coded samples was obtained from the University of California IRB (protocol 11-05519). Samples from Cameroon and Uganda screened by reverse transcriptase quantitative polymerase chain reaction (RT-qPCR) were described previously (Deng et al. 2020).

### HIV, hepatitis B virus, and hepatitis C virus serological and molecular screening methods

The testing algorithm for HIV, hepatitis B virus (HBV), and hepatitis C virus (HCV) has been outlined previously (Berg et al. 2021b), with exceptions made for specimens that did not have a sufficient volume for all assays. All specimens with a sufficient volume were screened with the Abbott ARCHITECT HBV surface antigen (HBsAg) Qualitative (4P53) assay and HIV Antigen/Antibody Combo (2P36) according to the manufacturer’s instructions. HIV viral load was quantified with the Abbott *m*2000 HIV-1 RealTi*m*e assay in serology-positive specimens. Specimens that were negative for HIV by serology and/or viral load testing were further screened with a research-use-only HIV-1/HBV/HCV multiplex polymerase chain reaction (PCR) assay conducted with the Abbott *m*2000sp (Abbott Molecular, Des Plaines, IL) and CFX96 Real Time (Bio-Rad, Hercules, CA) instruments in pools as previously described (Berg et al. 2021b). HBV viral load was quantitated with the *m*2000 HBV RealTi*m*e assay according to the manufacturer’s instructions for specimens that were positive for HBV in the multiplex assay. Likewise, HCV viral load was quantified with the *m*2000 HCV RealTi*m*e assay according to the manufacturer’s instructions for specimens that were positive for HCV in the multiplex assay. After the screening was completed, a panel of HIV-, HBV-, or HCV-positive, a mixture of coinfections, and HIV/HBV/HCV-negative specimens were selected for mNGS as summarized in the Supplementary Information, Table S1.

### NGS library preparation

Viral nucleic acids from samples were extracted using an EZ-1 Virus Mini Kit (Qiagen, Germantown, MD). Metagenomic libraries were prepared at the University of California San Francisco using the metagenomic sequencing with spiked primers (MSSPE) method as described previously (Deng et al. 2020). Briefly, thirteen-nucleotide spiked primers targeting the genomes of HIV, HBV, and HCV were added in a 10:1 ratio of spiked primers to random primers during the reverse transcription step of RNA library preparation, followed by cDNA library preparation using the Nextera XT kit (Illumina, San Diego, CA). Sequencing using dual-indexed barcodes was performed on an Illumina HiSeq instrument. The library containing Bangui orthobunyavirus, corresponding to sample CHBR594, yielded 1.88 million reads. To confirm the detection of Bangui orthobunyavirus, a 1:3 dilution of the remaining sample volume was pretreated with benzonase, followed by the total nucleic acid extraction on an *m*2000*sp* system (Abbott Molecular, Des Plaines, IL), reverse transcribed using SuperScript IV (ThermoFisher, Waltham, MA), converted into Nextera XT libraries, and sequenced on an Illumina MiSeq instrument at Abbott Laboratories as previously described (Berg et al. 2020).

### NGS data analysis

Demultiplexed FASTQ files were processed using the SURPI software pipeline, which utilizes SNAP (Zaharia et al. 2011) and RAPSearch (Zhao, Tang, and Ye 2012) to identify viral reads and an ABySS/Minimo pipeline (Simpson et al. 2009; Treangen et al. 2011) for *de novo* assembly. An additional in-house pipeline utilizing psiBLAST (Altschul et al. 1997) further characterized divergent viral reads. Contigs were evaluated with BLAST to ensure the correct assembly of ends, and full genome assembly was performed in CLC Genomics Workbench (Qiagen).

### Bunyavirus RT-qPCR assay

A multiplex RT-qPCR assay was developed on the Abbott *m*2000 system for the dual detection of the RdRp (FAM) and nucleocapsid (Cy5) genes of Bangui orthobunyavirus. Primers and probes were based on the CHRB594 sequence: RdRp Forward Primer: 5ʹ-ACCAGTAGCAAGTCCTGTATTA-3ʹ; RdRp Reverse Primer: 5ʹ-TTGCTATCTTACACTTCCTCTG-3ʹ; RdRp Probe oligo: 5ʹ-FAM-TGGCTGCTTTGAACTCTTGGAAATCACC-BHQ1-3ʹ; Nucleocapsid Forward Primer: 5ʹ-GCTTGGCCAACTCAGATTTAACA-3ʹ; Nucleocapsid Reverse Primer: 5ʹ-CTTTTCTTTCCACAGAGCAGCTT-3ʹ; and Nucleocapsid Probe oligo: 5ʹ-Cy5-CCGCATCTCCATGTTTGACTATGTCCC-BHQ2-3ʹ.

Target regions for assay development were cloned into pBlueScript and expressed from the T7 promoter using a MEGAscript T7 Transcription Kit (ThermoFisher, Waltham, MA) according to the manufacturer’s instructions. *In-vitro* transcripts targeting the L segment (RdRp) (BUABTRD; 597 nucleotides) and the S segment (nucleocapsid) (BUABTNC; 607 nt) were purified through Clontech Chroma Spin Column-100, size-confirmed on a 1.2 per cent formaldehyde agarose gel, and quantified by Qubit (ThermoFisher, Waltham, MA). A complete description of RT-qPCR assay volumes and cycling parameters for the *m*2000rt can be found in the Supplementary Information. For patient screening, plasma samples were pooled (1:3) and extracted on the *m*2000sp before the addition of quantitative polymerase chain reaction (qPCR) master mix and cycling on the *m*2000rt. Amplification curves were analyzed using MultiAnalyzer software.

### Sequence dataset retrieval and multiple sequence alignment

Bangui virus was initially identified as most closely related to Nyangole virus and viruses from members of the Anopheles A/B and Tanga/Okola serogroup using the BLAST algorithm. Two datasets containing complete genomes and associated metadata were downloaded from the National Center for Biotechnology Information nucleotide database on 7 March 2022 (https://www.ncbi.nlm.nih.gov/nucleotide/): the first (Dataset A) contained 703 orthobunyavirus representatives and the outgroup Khurdun virus and the second (Dataset B) contained 22 known viruses from the Anopheles A/B and Tanga serogroups with published genomes for all three segments. Whole-segment nucleotide alignments and protein alignments were performed using Clustal Omega (Sievers and Higgins 2018). The program *pal2nal* (Suyama, Torrents, and Bork 2006) was used to construct a codon-based nucleotide alignment of the open reading frames using the protein alignment and unaligned nucleotide coding sequences as input.

### Phylogenetic analyses

Maximum likelihood (ML) phylogenetic inference from the protein alignments was performed using IQ-TREE version 2.1.3 (Minh et al. 2020). The ModelFinder algorithm (Kalyaanamoorthy et al. 2017) was used to find the best-fitting substitution model according to the Bayesian Information Criterion score. The initial ML tree reconstruction was achieved using a stochastic algorithm and then optimized using the Nearest Neighbor Interchange heuristic method. The tree with the best log-likelihood score was retained, and branch and topology supports were provided using 1,000 replicates each of the Shimodaira–Hasegawa Approximate Likelihood Ratio Test and Ultrafast Bootstrapping (Anisimova and Gascuel 2006; Hoang et al. 2018). The phylogenetic information in Figs 2–4 was visualized using the *ggplot2* (Wickham 2016) and *ggtree* (Yu et al. 2017) tools in R, with the final figure annotation and layout performed on BioRender.com.

### Evaluation of temporal signal, calibration of molecular clock, and inference of evolutionary history

To assess the presence of a temporal signal (molecular clock) in the sequences of interest, two methodologies were followed. First, a root-to-tip analysis was used: ML trees and collection dates from Dataset B were provided as input to the programs TempEst and TreeTime to calculate an underlying temporal signal using the heuristic residual mean-squared and least-squares models, respectively (Rambaut et al. 2016; Sagulenko, Puller, and Neher 2018). Second, Bayesian Evaluation of Temporal Signal (BETS) (Duchene et al. 2020) was conducted using BEAST v.1.10.4 (Suchard et al. 2018) and the codon-based nucleotide alignments of the polyprotein from each viral segment from Dataset B. Briefly, BETS compares the Bayes factors (BFs) calculated from two different models, one in which the alignment is accompanied by the collection dates (heterochronous model) and the other in which the dates are omitted or considered contemporaneous (isochronous model). The estimation of the log marginal likelihood of these two models was achieved using path sampling (PS) (Lartillot, Philippe, and Lewis 2006) and stepping-stone sampling (SS) (Xie et al. 2011). Two clock models were tested: a strict clock and an uncorrelated relaxed lognormal clock (Drummond et al. 2006), together with two clock priors: an exponential distribution prior with a mean of 1.0 and a constant coalescent prior using an exponential population size with a mean of 10.0 and offset of 0.5. The analyses were run using BEAST 1.10.4 (Suchard et al. 2018) with a chain length of 5 × 10^8^ interactions, from which a 10 per cent burn-in was discarded. Thus, four total runs (one for each hypothesis with each clock prior) were conducted per genome segment. A comparison of the statistics from both the PS and SS methods was tabulated to compare the models (Table 1).

**Figure 1.**
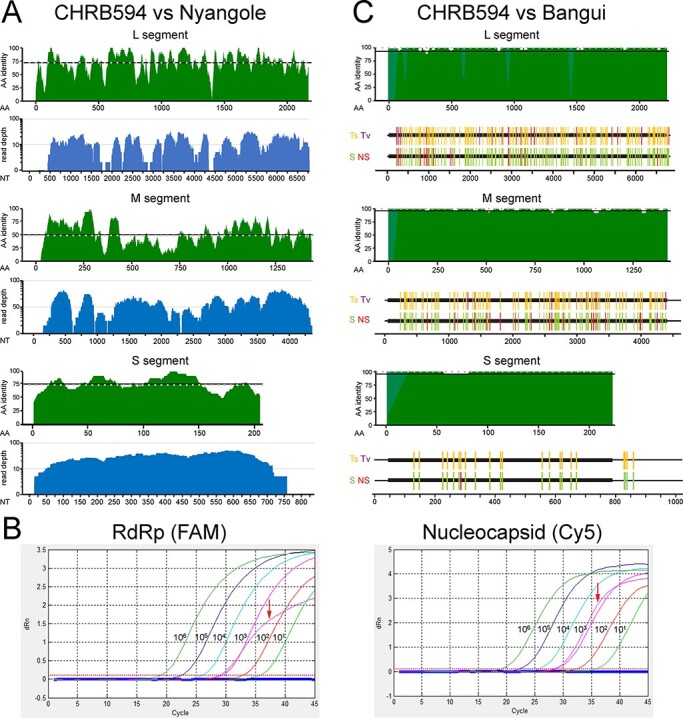
The independent discovery of a second Bangui virus strain. (A) Top (green): protein alignments performed between each genome segment of CHRB594 and Nyangole virus were scanned in twenty-five amino acid windows of the open reading frames. Dashed lines indicate the median per cent identity. Bottom (blue): NGS genome coverage plots for CHRB594. (B) RT-qPCR linearity for the L (left) and S (right) segments. The CHRB594 index case (bold curve) was detected in both channels at approximately *C_t_* = 28, corresponding to a viral load of 3.2 log copies per liter. (C) Top (green): protein alignments between each genome segment of CHRB594 and Bangui virus (accession numbers: MK896632.1, MK896631.1, and MK896630.1). Gaps in alignment from the missing sequence in CHRB594 were filled with light green. Bottom: nucleotide alignments of full-length sequences highlighting transitions (gold) and transversions (purple), silent mutations (green), and non-silent mutations (red) in CHRB594 relative to Bangui virus.

To infer the evolutionary history of Bangui virus, we combined Dataset B with the California serogroup, for which a time-stamped phylogeny and molecular rates have been previously described (Hughes et al. 2017) and re-estimated here: L = 5.875 × 10^−5^ substitutions/site/year; M = 6.789 × 10^−5^ substitutions/site/year; and S = 5.564 × 10^−5^ substitutions/site/year (Supplementary Information, Table S2). Thus, a time-measured evolutionary history was inferred using an Markov chain Monte Carlo analysis implemented in BEAST2 (Bouckaert et al. 2019). We employed two recently developed priors (BICEPS, with and without sampling dates, and Yule Skyline, without sampling dates) intended to better explain speciation rates of viral populations (Bouckaert and Rosindell 2022). We also used a nested sampling (NS) coalescent model (Skilling 2006; Russel et al. 2019) to determine the best fit to the dataset for both priors and two different clock models: strict and relaxed uncorrelated lognormal (Drummond et al. 2006). The quantification of the support for all the calculations was determined by conducting a BF test (Supplementary Information, Table S3). To provide statistical significance, we compared the log marginal likelihood estimationss obtained from the NS model evaluation and the SDs yielded for each prior and molecular clock used. The best-fitting prior (Yule Skyline) and molecular clock (relaxed uncorrelated lognormal) were used for phylogenetic reconstruction without sampling dates using BEAST2 with a chain length of 5 × 10^8^ interactions, from which a 10 per cent burn-in was discarded. Log files were evaluated using Tracer (Bouckaert et al. 2019), and convergence was considered achieved once the effective sample size parameter was greater than 250 for all major statistics. Maximum clade credibility (MCC) trees were generated by summarizing the 10,000 trees (minus 10 per cent burn-in) collected during each run using the program TreeAnnotator included with the BEAST2 package (Bouckaert et al. 2019).

All analyses were performed on a variety of high-performance Ubuntu-based computing resources using eight Tesla V100 16GB GPU cards (NVIDIA Corporation). The BEAGLE v.4.0 pre-release library (Ayres et al. 2012) and CUDA parallelization were used to enhance the computational speed.

### Evaluation of pervasive and episodic selection

To analyze the impact of both pervasive and episodic selection on the evolutionary process of the serogroups included in Dataset B, codon-based alignment of the polyprotein coding sequence of each viral segment and the corresponding isochronous MCC trees were used as input for the CODEML program of the PAML v.4.9 software package (Yang 2007).

The pervasive selection was estimated by using the site models M_2_ and M_8_ as described by Pikuła et al. (2021). Briefly, non-synonymous/synonymous mutation rate ratios (*dN/dS*, also called *ω*) were calculated for each site of the alignments, assuming a single average *ω* across the entire tree. The Bayes Empirical Bayes calculation of posterior probabilities for site classes was used to estimate the probabilities of sites under positive selection. As described previously (Perez et al. 2012), false positives were avoided by contrasting the models used to detect sites under positive pressure (M_2_ and M_8_) with models used to detect neutral selection (M_1_ and M_7_) (Anisimova and Yang 2007). Only cases in which the likelihood ratio test (LRT) result was significant were considered (Supplementary Information, Table S4). In addition, to test a discrete distribution of *ω* (also known as adaptation substitutions), an analysis contrasting models M_0_ and M_3_ was conducted (Yang, Wong, and Nielsen 2005).

The episodic selection was then assessed by branch-site models to determine the emergence of positively selected lineages, as described previously (Perez et al. 2022). Briefly, branch-site tests using prespecified branches, hypothesized to have emerged under positive selection (foreground branches) in comparison with the remaining branches (background branches), were tested with the alternative Model A. This model allows two classes of *ω* along all branches (0 < ω < 1 and ω = 1) and allows for an additional class only along prespecified foreground branches for codons under positive selection (ω > 1). This model was contrasted with the null Model A1, which does not allow ω > 1 along foreground branches (Yang 2007). The calculations were performed multiple times, each time selecting a particular clade as the foreground branch and the others as the background.

To conduct branch-site LRTs, the null model was chosen as a simplified version of the selection model with fewer parameters and was thus expected to provide a poorer fit to the data (lower ML). The significances of the LRTs were calculated assuming that twice the difference in the log ML between the two models was distributed as a *χ*^2^ distribution with the degrees of freedom assigned as the difference in the number of parameters between the two types of models (Supplementary Information, Table S5).

## Results

### An orthobunyavirus found in the DRC was originally isolated in the Central African Republic 50 years prior

After sequence-based characterization of HIV, HBV, and HCV in a large viral surveillance study conducted among 10,457 participants in Kinshasa, the DRC, from 2017 to 2019, a subset of 860 specimens were selected to be screened for viral pathogen discovery by mNGS. The subset included specimens testing negative for all three viruses and those that were singly or doubly infected (Supplementary Information, Table S1). mNGS screening was performed using the MSSPE ‘spiked’ primer approach that couples random priming with specific priming for HIV, HBV, and HCV viruses (Deng et al. 2020). The specimen CHRB594 was flagged for further inspection due to the detection of divergent bunyavirus reads from species in the *Orthobunyavirus* genus, including California encephalitis virus, Bunyamwera virus, Tacaiuma virus, Salt Ash virus, and Wyeomyia virus. This specimen was drawn on June 2017 from a 40-year-old male from Kinshasa. He was HBV positive (HIV/HCV negative), with an HBV viral load of 8.92 log copies/ml and an HBsAg signal to noise cut-off (S/CO) of 521.62. The full genome sequence of HBV from this patient was assembled and classified as genotype A.

Approximately 500 reads lacking nucleotide matches in GenBank were identified by the RAPSearch and psiBLAST algorithms as potential divergent hits to members of the *Orthobunyavirus* genus. The genome was assembled *de novo*, resulting in 94 per cent (706/13.7X), 96.2 per cent (562/17.3X), and 92 per cent (169/29.9X) coverage (reads/depth) for the L, M, and S segments, respectively. To rule out cross-contamination and confirm this discovery, an untouched aliquot of the original specimen was processed at a separate location (Abbott) using alternate extraction and library preparation methods and was successfully detected again by mNGS. For all three segments, the top BLAST hit was Nyangole virus, which was identified in 2019 (just prior to our discovery) in a febrile pediatric patient from Tororo, Uganda, diagnosed with malaria and pneumonia (Ramesh et al. 2019). At the amino acid level, it shared ∼72 per cent identity/86 per cent similarity for L (RdRp), ∼51 per cent identity/70 per cent similarity for M (glycoproteins), and ∼71 per cent identity/87 per cent similarity for S (nucleocapsid) with Nyangole virus (Fig. 1A). Consistent with other Anopheles A complex viruses (Mohamed, Mclees, and Elliott 2009), an alternate reading frame encoding the NS protein was absent on the S segment. In addition, the major nucleocapsid protein, N, extended ten amino acids beyond the 3ʹ end of other orthobunyaviruses.

We developed a research-use-only quantitative PCR assay based upon the index patient’s viral sequence for dual detection of the L (RdRp; FAM) and S (nucleocapsid; Cy5) segments with the goal of identifying additional viremic cases, comprehending genetic diversity, and assessing regional prevalence. Dose-dependent detection of *in-vitro* transcripts for both segments demonstrated assay linearity and a limit of detection of 10 copies/ml (Fig. 1B). For RdRp, a *C_t_* of 27.97 yielded a viral load of 3.10 log copies/reaction, while for nucleocapsid, a *C_t_* of 28.3 yielded a viral load of 3.29 log copies/reaction. Screening of 2,522 plasma specimens from individuals both positive and negative for HIV and/or HBV and collected in Cameroon, Uganda, and the DRC from years 2004 to 2019 failed to identify additional viremic (i.e. qRT-PCR-positive) infections.

In March 2021, the US Centers for Disease Control recovered the genomes of thirty-five previously un-sequenced bunyaviruses in their Arbovirus Reference Collection, isolated from vertebrates and arthropods over 66 years and from 27 countries (Kapuscinski et al. 2021). Most viruses had only been characterized serologically and through experimental infection of animals. Among the new orthobunyaviruses, Bangui virus (strain *DakHB 754*) appeared a near-identical match to the CHRB594 strain we identified in Kinshasa. At the protein level, amino acid identities for L, M, and S segments were 99.1 per cent (2,089/2,107), 98.7 per cent (1,385/1,403), and 99.5 per cent (214/215), respectively (Fig. 1C, top panel). The *DakHB 754* strain of Bangui virus was originally recovered in 1970 from the Central African Republic in a patient with fever and rash and was subsequently infected successfully in mice (Digoutte, Robin, and Cagnard 1973; El Mekki et al. 1981). While there still were numerous nucleotide differences between the two Bangui virus strains evenly distributed throughout each segment, very few gave rise to amino acid changes over the span of 50 years (Fig. 1C, bottom panels). For the L segment, there were 182/6,834 (2.66 per cent) mismatches overall (159 transitions and 23 transversions) compared to Bangui *DakHB 754*. In the coding regions, most mutations (156; 89 per cent) were silent, and only eighteen were non-synonymous. There were 113/4,623 (2.44 per cent) nucleotide mismatches (106 transitions and 7 transversions) in the M segment of CHRB594 compared to Bangui *DakHB 754*. Again, most mutations (95; 84 per cent) were silent, with only eighteen non-synonymous mutations. Finally, for S, there were 22/1,023 (2.15 per cent) nucleotide mismatches (22 transitions and 0 transversions) compared to Bangui *DakHB 754*. Every mutation in the nucleocapsid open reading frame except one was silent. Thus, we identified a second strain of Bangui virus nearly 50 years after the original isolate.

### Bangui virus clusters with Nyangole virus to form a distinct clade within a group containing the Tanga, Anopheles A, and Anopheles B serogroup complexes

**Figure 2.**
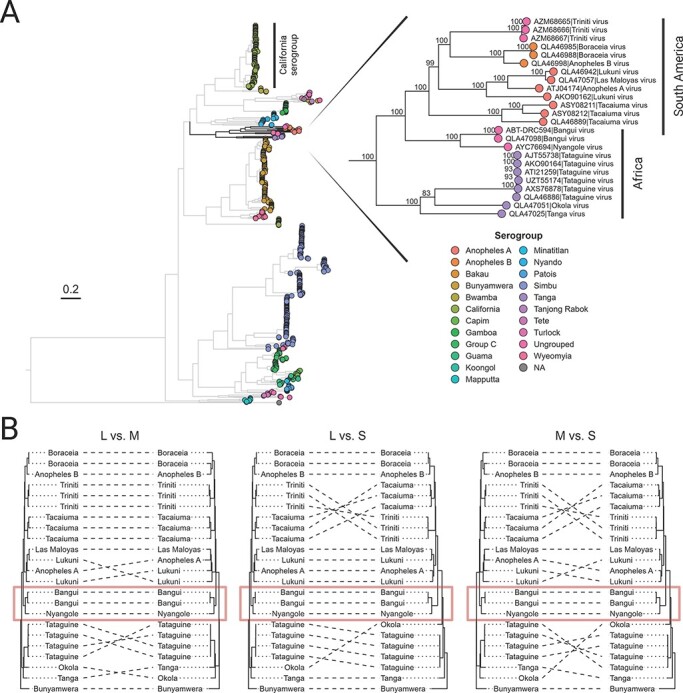
ML reconstruction of the *Orthobunyavirus* genus, indicating the position of Bangui virus. (A) A total of 703 orthobunyavirus L segment polyprotein amino acid sequences are represented. The tree is outgroup-rooted on Khurdun virus, an unclassified member of the *Peribunyaviridae* family. The tips are color-coded based on the established or assumed serogroup. The monophyletic group containing the Tanga serogroup, Anopheles A/B serogroups, and Bangui–Nyangole clade is magnified on the right. Bangui and Nyanogole viruses cluster together as a monophyletic group within the African clade, basal to the Anopheles A serogroup. Branch supports calculated using 1,000 replicates of ultrafast bootstrapping are shown at the nodes in the magnified portion of the tree. Analogous trees for the M and S segments can be found in the Supplementary Information, Fig. S1. (B) A collection of tanglegrams comparing the ML trees computed from the polyprotein amino acid sequence from each segment of a reduced set of twenty-three closely related orthobunyaviral genomes with published sequences for all three segments. Each tree in panel B is rooted using Bunyamwera virus as the outgroup. The tanglegrams illustrate putative reassortment events by highlighting when two segments from the same viral isolate appear in different configurations between their respective ML trees.

An ML tree reconstruction using amino acid sequences available for 721 orthobunyavirus isolates showed a well-supported clustering pattern for the L segment of Bangui virus with Nyangole virus in a serologically unclassified clade (henceforth called the ‘Bangui–Nyangole clade’) (Fig. 2A). The Bangui–Nyangole clade groups within the Anopheles A/B and Tanga serogroup complexes from South America and Africa, respectively, and branches near the division of those groups. This branching pattern is also observed for the glycoprotein and nucleocapsid although this can be influenced by the specific taxa included in the analysis (Supplementary Information, Fig. S1). This cluster of serogroups shows long branch lengths and less diversification than other serogroups known for causing outbreaks in humans such as California (e.g. La Crosse Virus) or Simbu (e.g. Oropouche).

To gain resolution for the clustering and branching distances between the viral species closest to Bangui virus, we focused on twenty-two viruses in the Tanga and Anopheles A/B serogroups (Fig. 2A, inset), for which sequences of all three segments are available, and re-estimated the phylogenies based on ML inference of a codon-based nucleotide alignment (Fig. 2B). The resolved topologies supported the same pattern for all three segments of Bangui virus, in that it consistently forms a monophyletic group with Nyangole virus, regardless of whether the alignments were based on nucleotides or amino acids (Supplementary Information, Figs S2 and S3). Despite the two Bangui virus strains being collected nearly 50 years apart, the higher-resolution ML trees reveal a lack of genetic distance at the amino acid level (Supplementary Information, Fig. S3) and only a small distance at the nucleotide level (Fig. 2B, Supplementary Information, Fig. S2). These results collectively revealed that minimal diversification has occurred between these two strains.

We also observed genomic reassortment for some viral species within this group as a potential source of genetic diversity. For example, the M segment of Anopheles A virus branched most closely to Las Maloyas virus, whereas in the L and S segments, it branched most closely to Lukuni virus (Fig. 2B). Additionally, the S segment of Tacaiuma virus and the serologically unclassified Triniti virus showed evidence of reassortment, while reassortment of the M segment was seen within the four sequenced strains of Tataguine virus. In contrast, reassortment was not observed for the Bangui–Nyangole clade.

### The lack of evolutionary temporal signal indicates an ancient emergence of viruses in the Bangui–Nyangole clade

To further explore the evolutionary history of both Bangui virus strains within the context of the Tanga and Anopheles A/B serogroups, molecular clock analyses were performed. Root-to-tip distance regression of the ML trees using TempEst and TreeTime failed to establish a molecular clock signal (Supplementary Information, Fig. S4). BETS using codon-based nucleotide alignments was then performed with marginal likelihoods estimated using both PS and SS. Both PS and SS methods showed stronger support for isochronous (time-independent) topologies compared to heterochronous (time-dependent) topologies, further indicating that these viruses do not conform to a molecular clock (Table 1).

**Figure 3.**
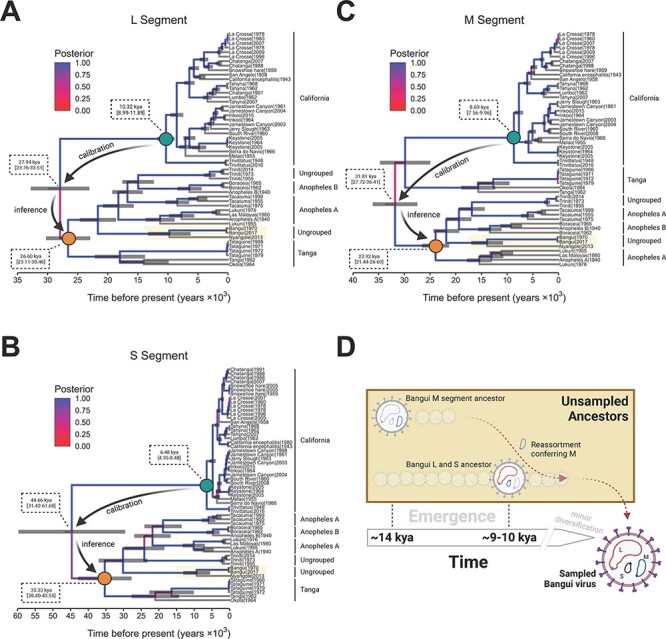
The temporal reconstruction of the emergence of Bangui virus and the closest phylogenetically related viral species. (A–C) MCC trees based on the three genomic segments (L, M, and S) for all the viral species evaluated. Each time-scaled phylogeny resulted from the calibration of the evolutionary rate using the California serogroup viruses. The branches are colored according to posterior probability, and median ages with 95 per cent HPD confidence are shown at ancestral nodes. The process utilized to infer the ancestrality of the Tanga/Anopheles serogroup (orange dot) through calibration using the California serogroup (green dot) is highlighted in each tree. (D) The evolutionary model depicting the ancient reassortment and mutation events that resulted in the emergence of the sampled Bangui virus.

Due to the lack of temporal signal obtained from the Tanga/Anopheles groups in isolation, we addressed the inference of evolutionary history by calibrating the molecular clock using the closely related California serogroup (Fig. 2A), for which evolutionary emergence has been previously reported (Hughes et al. 2017) and rates were inferred (Supplementary Information, Table S2). An evaluation of priors recently developed to reconstruct speciation rates of viral populations (BICEPS and Yule Skyline) (Bouckaert and Rosindell 2022) favored Yule Skyline with a relaxed uncorrelated lognormal clock (Supplementary Information, Table S3). Using the California serogroup’s evolutionary rates as a calibrator allowed us to estimate the time of the most recent common ancestor (tMRCA) of the root of the tree and the evolutionary rates for each segment (Fig. 3 and Supplementary Information, Table S4). We inferred that the tMRCAs for the Tanga/Anopheles L and S segments were ∼26,000 years ago and ∼35,000 years ago, respectively (Fig. 3A and B). The M segment appears to have a different evolutionary history, as evidenced by the Tanga and Anopheles group splitting at the root, approximately 33,000 years ago (Fig. 3C). Considering that the 95 per cent height posterior density (HPD) confidences of these emergence times overlap, we can estimate that the Tanga/Anopheles group emerged about 20,000 years before the last common ancestor of the California serogroup.

With this timescale established, the ancestor of the Bangui–Nyangole clade’s M segment diverged with a median time of ∼14,000 years ago (Fig. 3C). The emergence of this clade’s L and S segments occurred closely together with their ancestors diverging with median time between 9,500 and 10,000 years ago (Fig. 3A and B). Given the overlap of the 95 per cent HPD confidences, those events could have taken place at the same time, yet we call attention to the different evolutionary history of the M segment (Fig. 3C), wherein the division between African and South American strains is unclear. Summarizing these findings suggests a model in which the L and S segments co-evolved and acquired an M segment through reassortment to result in the modern Bangui/Nyangole clade (Fig. 3D). For historical context, the time periods described previously correspond to the rise of farming and larger human civilization in central Africa during the Neolithic era.

### Negative purifying selection constrains host-jumping events and diversification of Bangui and related viruses

Two fundamental questions arose when considering the emergence of Bangui virus to be ∼9,500 years ago: (1) why has Bangui virus gone unnoticed for nearly 50 years? and (2) why does it appear to not actively circulate in the human population? Bangui virus and its close relatives Nyangole, Tataguine, and Tacaiuma have been documented to induce acute fever, rash, and arthralgia in humans (Digoutte, Robin, and Cagnard 1973; Calisher et al. 1980; Ramesh et al. 2019; Simo Tchetgna et al. 2019). Additionally, the Ntwetwe virus (not shown in our phylogenetic trees since only a partial genome is available) has been linked to fatal encephalitis (Edridge et al. 2019). Other close relatives (e.g. Boraceia, Las Maloyas, Okola, and Tanga viruses) have only been isolated from mosquitos, so it is unclear whether these can efficiently transmit to the human population and cause diseases. Therefore, to understand the evolutionary forces influencing the diversification and persistence of the entire viral group (and thus spillover potential), both pervasive and episodic selection pressures were investigated. Site model and branch-site model algorithms, implemented in the program CODEML, were used to estimate synonymous substitution rates (*dS*), non-synonymous substitution rates (*dN*), and the ratio between them (*dN*/*dS*, also called *ω*). These values were assessed both between sites and between branches from the estimated temporal topologies.

**Figure 4.**
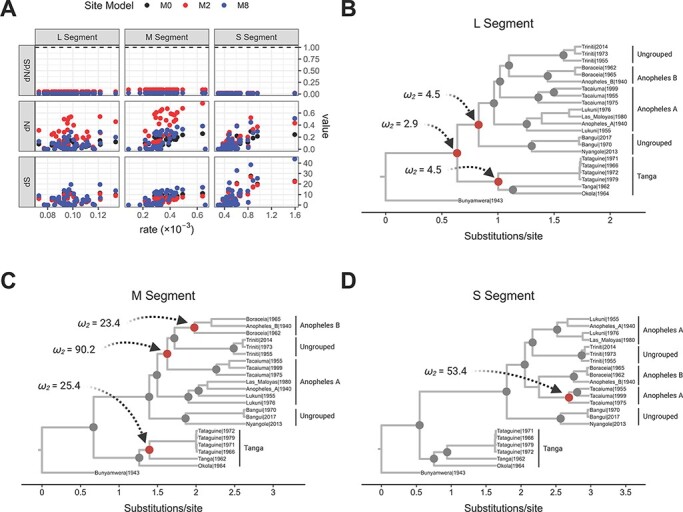
The action of episodic and pervasive selection on Bangui virus and closest phylogenetic viral species. (A) Values of synonymous (*d_S_*) and non-synonymous (*d_N_*) mutation rates (y-axis) inferred by CODEML plotted against substitution rates inferred by BEAST (x-axis). Each dot represents a single node and is colored according to the site model (estimation of pervasive selection) used for the calculation within CODEML. (B–D) Isochronous Bayesian MCC trees based on the three segments of Bangui virus and the closest-related viral species with selection events calculated using the branch-site models (episodic selection) in CODEML. All the diversification nodes were tested individually with contained branches classified as foreground branches. Nodes colored gray yielded a *ω* < 1 when the daughter branches were selected as foreground branches, while nodes colored red yielded a *ω* > 1 when the daughter branches were selected as foreground. In all cases, *ω_2_* refers to Class 2a of the foreground branches.

Based on the Bayesian posterior probabilities determined by the M2 and M8 site models, no codon sites in the L, M, and S segments were found to be under positive selection pressure (Fig. 4A and Supplementary Information, Table S5). For all three segments, numerous nucleotide mutations accumulated over many years for all viral species analyzed (Fig. 4A, bottom panels indicating *dS*); however, most of those mutations did not generate changes in the corresponding amino acid residues (Fig. 4A, middle panels, *dN*). Hence, all values of *ω* (*dN/dS*) were lower than 0.25 (Fig. 4A, upper panels), indicating that, for the entire group of viruses analyzed, mutations on each segment that lead to amino acid changes were being acted upon by purifying selection (*ω* < 1).

We next asked whether Bangui and related viruses were under episodic selection while circulating in their host, thereby conferring the potential to cause an outbreak in the human population. To determine this, we explored the *ω* at each bifurcation in a non-time-stamped Bayesian MCC tree for each segment, again using the branch-site models implemented in CODEML. We observed that the earliest diversification events in ancestors of the entire Tanga/Anopheles group carrying the L segment were driven by positive selection; however, none of the subsequent diversification in L appears to be (Fig. 4B and Supplementary Table S6). Three diversification events in the M segment were observed to have been positively selected. These events led to the divergence of the Tacaiuma virus, Triniti virus, and the Anopheles B serogroup away from other Anopheles A serogroup members, as well as the emergence of the Anopheles B and Tanga serogroups (Fig. 4C and Supplementary Information, Table S5). In contrast, only one positively selected diversification event was observed in the S segment: the emergence of the Tacaiuma virus in South America (Figs 4D and 3C and Supplementary Information, Table S6). Notably, the last common ancestor for all segments of the Nyangole virus and Bangui virus did not emerge due to positive selection. Therefore, the episodic selection also did not explain their speciation, suggesting that these species may have emerged from a host-jumping event that was not evolutionarily favored.

**Table 1.** Bayesian evaluation of temporal signal for each bunyaviral genome segment. The *log marginal likelihood* values were estimated using either path sampling or stepping-stone sampling methods. The *log Bayes factor* value is computed as the difference between the *log marginal likelihood* values estimated using sampling dates (heterochronous model) and omitting sampling dates (isochronous model).

	Clock prior: exponential distribution, mean = 1.0						Clock prior: constant coalescent using an exponential population size, mean = 10.0 and offset = 0.5					
	Path sampling			Stepping-stone sampling			Path sampling			Stepping-stone sampling		
	L	M	S	L	M	S	L	M	S	L	M	S
Log marginal likelihood, isochronous model	−97,168.63	−78,848.36	−11,725.34	−97,168.65	−78,848.44	−11,725.47	−97,388.53	−78,995.26	−11,713.03	−97,388.19	−78,994.78	−11,712.99
Log marginal likelihood, heterochronous model	−97,193.26	−78,877.52	−11,748.24	−97,193.35	−78,877.89	−11,748.48	−97,447.07	−79,047.00	−11,757.16	−97,446.28	−79,046.05	−11,756.95
Log Bayes factor	−24.63	−29.16	−22.90	−24.70	−29.45	−23.01	−58.54	−51.74	−44.13	−58.09	−51.27	−43.96
Temporal signal	No	No	No	No	No	No	No	No	No	No	No	No

## Discussion

The novel bunyavirus we sequenced from the Democratic Republic of the Congo in 2019 was originally found in 1970 in the Central African Republic and named Bangui virus (Digoutte, Robin, and Cagnard 1973; El Mekki et al. 1981; Kapuscinski et al. 2021). Given that this is only the second reported sequence in more than five decades, it appears as if Bangui virus infection in humans is rare, with minimal reservoir spillover and likely no human-to-human transmission (Mayanja et al. 2021). The individual from whom our strain of Bangui virus was isolated had a high-titer HBV coinfection, and, unfortunately, we lacked the clinical information to indicate whether he presented with symptoms beyond those consistent with hepatitis. Our efforts to identify additional infections in Africa by qPCR (*n* = 2,522) and mNGS (*n* = 859) were unsuccessful, yet not altogether surprising given the results of our phylogenetic analysis. Of the >170 viruses in the *Orthobunyavirus* genus, only ∼30 are known to cause diseases in mammals and result in outbreaks, such as LaCrosse, Oropouche, and Schmallenberg viruses (Elliott 2014). A large percentage of bunyaviruses (and arboviruses in general) are maintained through horizontal and vertical transmission in strict insect vector ranges, thereby removing the requirement for a mammalian host (Beaty and Calisher 1991). Thus, acute, but rare, self-limiting bunyavirus infections in humans seem to be the rule rather than the exception (Elliott and Weber 2009). Examples range from acute febrile illness and arthralgia in Bangui virus infections to severe encephalopathy from several other orthobunyaviruses, including Nyangole, Tacaiuma, and Ntwetwe viruses (de Melo Junior et al. 2018; Edridge et al. 2019; Ramesh et al. 2019). While members of the Anopheles A/B and Tanga serogroups all appear to be vectored by mosquitos, there is no phylogenetic delineation with regard to which viruses can cause diseases in humans. Although the Bangui–Nyangole clade has not been serologically classified, it likely represents a novel serogroup based on the long phylogenetic distances between the clade and related serogroups (Figs 2–4); further studies are needed to confirm this.

Based on the genetic relationships between the different viral strains in the Tanga/Anopheles serogroups (Fig. 2 and Supplementary Fig. S1), all three genome segments showed similar branching topologies for Bangui virus, excluding reassortment as an explanation for its emergence. These results, based on ML inferences, contrast with those from a recent study by Kapuscinski et al. (2021), which presented the M segment of Bangui branching most closely with Tacaiuma virus, suggesting a South American origin for Bangui’s M segment. We believe that this discrepancy is the consequence of the omission of Nyangole virus from that study’s dataset since we replicated their findings when we removed Nyangole virus (Supplementary Information, Fig S1A, inset). On the other hand, their use of Bayesian inference for topological reconstruction could have also influenced these differences.

Interestingly, our Bayesian temporal analysis (Fig. 3) shows that the emergence of the M segment of Bangui and Nyangole viruses may have been derived from a South American ancestor. It also reveals that the L and S segments of these bunyaviruses share the same evolutionary history. This incongruence between the emergence time of the M segment versus that of L and S has been frequently observed by others, suggesting that the mechanisms driving the emergence of novel bunyaviruses are characterized by reassortment events. Several reports illustrate this trend, such as the emergence of Ngari virus resulting from a reassortment of the L and S segments from Bunyawerma and M from Batai virus (Briese et al. 2006). Likewise, the emergence of the Iquitos, Madre de Dios, and Perdoes viruses is the result of the reassortment of the L and S segments from Oropouche virus and the M segment from a novel Simbu serogroup virus (Aguilar et al. 2011; Ladner et al. 2014; Tilston-Lunel et al. 2015; Navarro et al. 2016).

Although the number of sequences used in the current study limited the estimation of phylogeographic structure among the different viral strains, we can infer that a split between African and South American strains occurred roughly 22,000–30,000 years ago, based on the evolutionary history of the L and S segments (Fig. 3A and B). We believe that the emergence of Bangui occurred approximately 10,000 years ago (Fig. 3D), a timeframe consistent with the presence of the Beringian land bridge connecting Asia and North America during the last glacial maximum that allowed for significant migration of animals and peoples to the Americas (Goebel, Waters, and O’Rourke 2008; Hu et al. 2010). This period has also been linked to the emergence of several viral species in the Americas, including HTLV-2 (Lemey et al. 2005), Powassan virus (Gould et al. 2003), and several other flavivirus species (Moureau et al. 2015).

For Bangui and related viruses, the molecular clock hypothesis was tested and rejected, and a minimal accumulation of genetic diversity was observed over time. As a result, the substitution rates observed for this entire group were on the order of 10^–5^ substitutions/site/year, contrasting with contemporaneously evolving orthobunyaviruses such as Oropouche virus that display evolutionary rates on the order of 10^–3^ substitutions/site/year (Gutierrez et al. 2020). This finding categorizes Bangui as a ‘slowly evolving’ RNA virus (Dolan, Whitfield, and Andino 2018). The fact that Bangui virus seems to evolve 10–100 times more slowly compared to other well-known arboviruses could be a consequence of the strains sequenced from both human cases being the direct outcome of rare spillover events. Thus, the evolutionary rates reflect the effect of both this virus being in equilibrium with its natural host and the additional constraints caused by the immune response of the vector.

**Figure 5.**
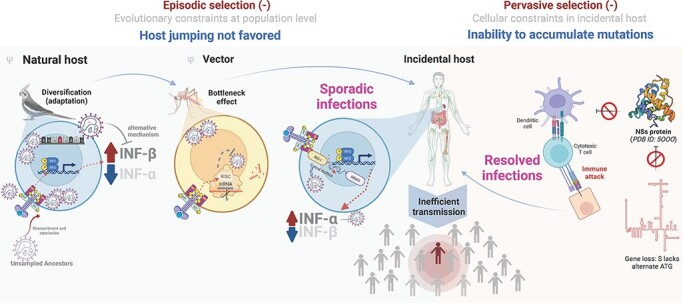
The schematic representation of events driving Bangui virus toward evolutionary constraint and inefficient spread in the human population. Left panel: a candidate model for how episodic selection may have affected the evolutionary trajectory of Bangui virus from its putative natural host (bird) to its vector (mosquito) and incidental host (human). ^Ψ^ denotes the hypothesized natural host (Dégallier et al. 1992; Livonesi et al. 2007; Mohamed, Mclees, and Elliott 2009; Qu et al. 2013), and ^φ^ denotes the hypothesized vector (Léonard et al. 2006; Baxter, Contet, and Krueger 2017; de Melo Junior et al. 2018) based on the circulation of representative based on the findings from phylogenetically close representative viral members. Right panel: a candidate model for how pervasive purifying selection acts on Bangui virus to limit the transmission and circulation in the human population.

Despite both the natural host and vector being unknown for Bangui virus, we can formulate some hypotheses based on phylogenetic and viral data (Fig. 5). First, as Bangui virus groups in an intermediate clade branching with the Anopheles A/B serogroups, the most probable vector for this virus is an *Anopheles* mosquito species. Mosquito species keep viral infections latent, but constrained, by the immune response triggered by Toll receptors via silencing RNA interference (RNAi) (Baxter, Contet, and Krueger 2017) or the JAK-STAT/Vago pathways (Souza-Neto, Sim, and Dimopoulos 2009; Paradkar et al. 2012). Thus, despite the presence of uncharacterized JAK-STAT orthologs in *Anopheles spp.* (Souza-Neto, Sim, and Dimopoulos 2009), the RNAi mechanism is present, is active, and likely constrains the Bangui viral population within this vector. These mechanisms apply selective pressure on the viral population and act as a genetic bottleneck (Fig. 5, left).

It has been observed experimentally that some members of the Anopheles serogroups can suppress host interferon (IFN)-β response (Mohamed, Mclees, and Elliott 2009) although they are still partially susceptible to IFN-α (Livonesi et al. 2007). Thus, the natural host for Bangui virus, as for other members of the phylogenetic group studied here, is likely to possess a differential level of IFN-α/β in the innate immune response, with a lower level of IFN-α (that Bangui virus is susceptible to) and a higher IFN-β level (that Bangui virus can evade). Birds exhibit this profile; chickens have recently been reported to differentially express type I IFNs opposite to that of mammals (i.e. higher levels of IFN-β than IFN-α) as a response to multiple kinds of viral infections such as infectious bursal disease and influenza (Qu et al. 2013). Additionally, a high prevalence of antibodies against Tacaiuma virus has been reported in several bats, small rodents, and birds, suggesting that these species may act as amplifying hosts (Calisher et al. 1980; Karabatsos 1985; Dégallier et al. 1992; Dias et al. 2022). These findings suggest the likelihood of a natural avian reservoir for Bangui virus. Through an ability to evade IFN-β (Livonesi et al. 2007; Mohamed, Mclees, and Elliott 2009) and with lower levels of host IFN-α expression, the Bangui viral population is kept in equilibrium within this putative avian host and is allowed to accumulate mutations. By contrast, sporadic infections of Bangui virus in the incidental human host are confronted by an innate immune response characterized by high levels of IFN-α and are readily cleared. Thus, antiviral responses constrain viral population diversification, indicating that episodic selection acts as a purifying force for Bangui. It is relevant to note, however, that avian type I IFNs are positioned at the root of the phylogenetic tree of mammalian type I IFNs, suggesting that the role of type I IFN between these two groups may be reasonably different (Shaw et al. 2017). Environmental sampling and experimental studies will be required to determine whether Bangui virus has an avian reservoir.

The pervasive selection also acts on Bangui virus as a purifying force limiting the transmission and circulation in the incidental human host (Fig. 5, right). Like other members of Anopheles A/B and Tanga serogroups, Bangui virus lacks the alternative initiation codon for the non-structural NS protein encoded by the S segment in other orthobunyaviruses (Mohamed, Mclees, and Elliott 2009). This viral protein acts as an IFN-antagonist to thwart the host’s innate immune responses (Léonard et al. 2006). Two reports have shown that the NS protein blocks the transcription of type I IFN genes (Weber et al. 2002), including IFN-α and IFN-β, through interaction with the cellular factor *Med8* (Thomas et al. 2004; Léonard et al. 2006), thus allowing for evasion of the host’s innate immune responses. Without this evasion mechanism, the virus is likely to be less able to prevent the adaptive response (e.g. activating cytotoxic cells and recruiting B-cells). Nonetheless, it has been observed experimentally that some members of the Anopheles serogroups can still partially suppress IFN-β response from an alternative mechanism not involving NSs (Mohamed, Mclees, and Elliott 2009). While the presence of an NS gene alone does not predict orthobunyaviral pathogenicity in humans, it is interesting to note that serogroups observed or suspected to have an avian host (i.e. Tete and Guama) all lack the NS gene like Bangui virus (Shchetinin et al. 2015).

East and Central African nations have a rich history of Orthobunyaviral isolation and identification by non-sequencing techniques since at least 1930. Isolated or repeated Batai, Bunyamwera, Bwamba, Germiston, Ilesha, Ngari, Nyando, and Pongola virus outbreaks have been documented in Central African Republic, Cameroon, Uganda, Kenya, Tanzania, Sudan, and Somalia (Braack et al. 2018; Nyaruaba et al. 2019). Thus, the limited viral genomes available for the analysis within the serogroups of interest (Tanga/Anopheles) are probably not the result of undersampling in the human population. The long phylogenetic branch lengths separating the viral species within the serogroups of interest, contrasted by the short branch lengths separating the two Bangui strains (despite being isolated 50 years apart), illustrates diversification within the natural host without the acquisition of the necessary fitness to adapt to the human host (Grubaugh et al. 2019). We conclude that, for the viral strains sampled and perhaps for unsampled ancestors waiting to be discovered, speciation events within the host or vector are not being driven by positive, episodic selection. Indeed, the latest event driven by the action of the positive selection in the serogroups analyzed here occurred ∼6,000 years ago with the diversification of the S segments of Tacaiuma virus. Specific to Bangui virus, the only detected event favored by episodic selection seems to have occurred during the diversification of the last common L segment ancestor of the Bangui/Nyangole, Anopheles A, Anopheles B, and Triniti clades. This was an event that preceded the acquisition of the modern M segment.

## Conclusions

Not all zoonotic viruses warrant the same level of attention. Here, we show that the genetic composition of a Bangui virus strain isolated 50 years ago is essentially the same as the one collected in the present day; coupling the lack of anti-host defenses, the dearth of variant strains, and the absence of recent positive selection events, we believe that Bangui virus and others related to it are hindered from causing active outbreaks in the human population. The phylogenetic approaches shown here may provide the tools to predict whether a virus is of little concern or represents an emerging threat. This has a great value from the standpoint of public health and whether to invest in developing targeted molecular and serologic assays for the virus. As metagenomic approaches lead to the discovery and characterization of an increasingly large number of new viruses, applying analyses like those described here will be essential to inform which viruses merit such concern.

## Supplementary Material

vead018_Supp

## Data Availability

R scripts used to generate figures are available on Github at https://github.com/gregory-orf-phd. The CHRB594 sequences are available at GenBank under the accession numbers OP941175, OP941176, and OP941177.
