# Supplementary material for: Purifying selection decreases the potential for Bangui orthobunyavirus outbreaks in humans
Source: Virus Evol. 2023 Mar 8;9(1):vead018. doi: 10.1093/ve/vead018 (PMC10072187; doi:10.1093/ve/vead018)
Supplement: vead018_Supp [file vead018_supp.zip › suppl_data/Detection of Bangui virus in DRC_SI_v6_clean.docx]

**Supplementary Information for:**

**Purifying selection decreases the potential for Bangui orthobunyavirus outbreaks in humans**

**Gregory S. Orf**^1,‡^, **Lester J. Perez**^1,‡^, Todd V. Meyer^1^, Ka-Cheung Luk^1^, Kenn Forberg^1^, Mary A. Rodgers^1^, Abbas Hadji^1^, Linda James^2^, Samuel Mampunza^2^, Asmeeta Achari^4^, Guixia Yu^4^, Scot Federman^4^, Charles Y. Chiu^4,5^, Carole A. McArthur^3^, Gavin A. Cloherty^1^, Michael G. Berg^1,^*

^1^ Abbott Laboratories and Abbott Pandemic Defense Coalition, Abbott Park, IL, USA

^2^ Université Protestante au Congo, Kinshasa, Democratic Republic of Congo

^3^ University of Missouri-Kansas City, Kansas City, MO, USA

^4^ Department of Laboratory Medicine, University of California San Francisco, San Francisco, CA, USA.

^5^ Department of Medicine, Division of Infectious Diseases, University of California San Francisco, San Francisco, CA, USA.

**^‡^ equal contribution**

* Corresponding author: Michael G. Berg, [michael.berg@abbott.com](mailto:michael.berg@abbott.com)

**SUPPLEMENTAL METHODS**

**Table S1**. Panel of HIV+, HBV+, HCV+, a mixture of coinfections and HxV- specimens from the Democratic Republic of Congo selected for mNGS. Note, 220 samples were reserved after HIV testing for sequencing due to low volume and were not screened for HBV and/or HCV.

| HIV | HBV | HCV | N |
| --- | --- | --- | --- |
| Negative | Negative | Negative | 51 |
| Negative | Negative | Positive | 10 |
| Negative | Positive | Negative | 78 |
| Positive | Negative | Negative | 41 |
| Negative | Positive | Positive | 2 |
| Positive | Negative | Not tested | 423 |
| Positive | Positive | Not tested | 32 |
| Positive | Not tested | Not tested | 192 |
| Negative | Not tested | Not tested | 28 |
| Positive | Positive | Negative | 3 |

**Bunyavirus RdRp/NC duplex quantitative PCR Reaction (50 µl per rxn)**

1. Master mix for 1 reaction (final reaction volume, 50 µl):

**Component Volume Final Conc.**

Water 10.9 µl

2 x RT-PCR Buffer 25.0 µl (1X)

BUFP (RdRp) (100 µM in TE, pH8.0) 0.2 µl (0.4 µM)

BURP (RdRp) (100 µM in TE, pH 8.0) 0.2 µl (0.4 µM)

BUNCFP (NC) (100 µM in TE, pH 8.0) 0.2 µl 0.95 µl (0.4 µM)

BUNCRP (NC) (100 µM in TE, pH 8.0) 0.2 µl (0.4 µM)

BUPRO (FAM) (RdRp) (100 µM in TE, pH 8.0) 0.15 µl (0.3 µM)

BUNCPRO (Cy5) (NC) (100 µM in TE, pH 7.0) 0.15 µl (0.3 µM)

50 mM MgCl_2_ 1.0 µl (1 mM)

25 x RT-PCR Enzyme Mix 2.0 µl (1X)

**Total 40 µl**

1. Dispense master mix into plate and add RNA:

Master mix 40 µl

RNA 10 µl

**Total 50 µl**

1. The following thermocycle is performed:

| Stage | Cycle | Temperature (ºC) | Time (min) |
| --- | --- | --- | --- |
| 1 | 1 | 50 | 30 |
| 2 | 1 | 95 | 10 |
| 3 | 45 | 95 | 0.5 |
|  |  | 62 | 0.5 |
|  |  | 55 | 1.5 (read signal in the last 0.5) |

**Notes:**

1. Forward primer (BUFP for RdRp), reverse primer (BURP for RdRp), FAM probes (BUPRO for RdRp), forward primer (BUNCFP for NC), and reverse primer (BUNCRP for NC) are all resuspended in TE, pH 8.0, to 100 µM. The following volumes are mixed in one tube: 100 µl BUFP for RdRP, 100 µl BURP for RdRP, 75 µl BUPRO for RdRP, 100 µl BUNCFP for NC, and 100 µl BUNCRP for NC. Just add 0.95 µl of this mixture for each 50 µl reaction. The tube has 475 µl which is enough for 500 reactions.
2. BUNCPRO Cy5 probe targeting CHRB594 in S/nucleocapsid (NC) is resuspended in TE, pH 7.0, to 100 µM. Just add 0.15 µl of the Cy5 probe for each 50 µl reaction. The tube has 75 µl which is enough for 500 reactions.
3. *In vitro* transcripts: BUABTRD (CHRB594 strain L/RdRp) and BUABTNC (CHRB594 strain S/nucleocapsid) are mixed in one tube and provided in water at 10^6^, 10^5^, 10^4^, 10^3^, 10^2^, 10^1^ copies/10 µl. That is, each 10 µl has 10^6^, 10^5^, 10^4^, 10^3^, 10^2^, 10^1^ copies of each of the two transcripts. Each concentration has 4 x 90 µl aliquots. Store transcripts at -70 ºC.
4. ROX is a reference dye in 2 x RT-PCR buffer.
5. 1 ml of 50 mM MgCl_2_ (Sigma) is provided as an activator.
6. AgPath-ID One-Step RT-PCR Kit (Life Technologies, cat# 4387424) includes 2 x RT-PCR Buffer, 25 x RT-PCR Enzyme Mix, Detection Enhancer (x15) and Nuclease-free water.

**SUPPLEMENTAL RESULTS**


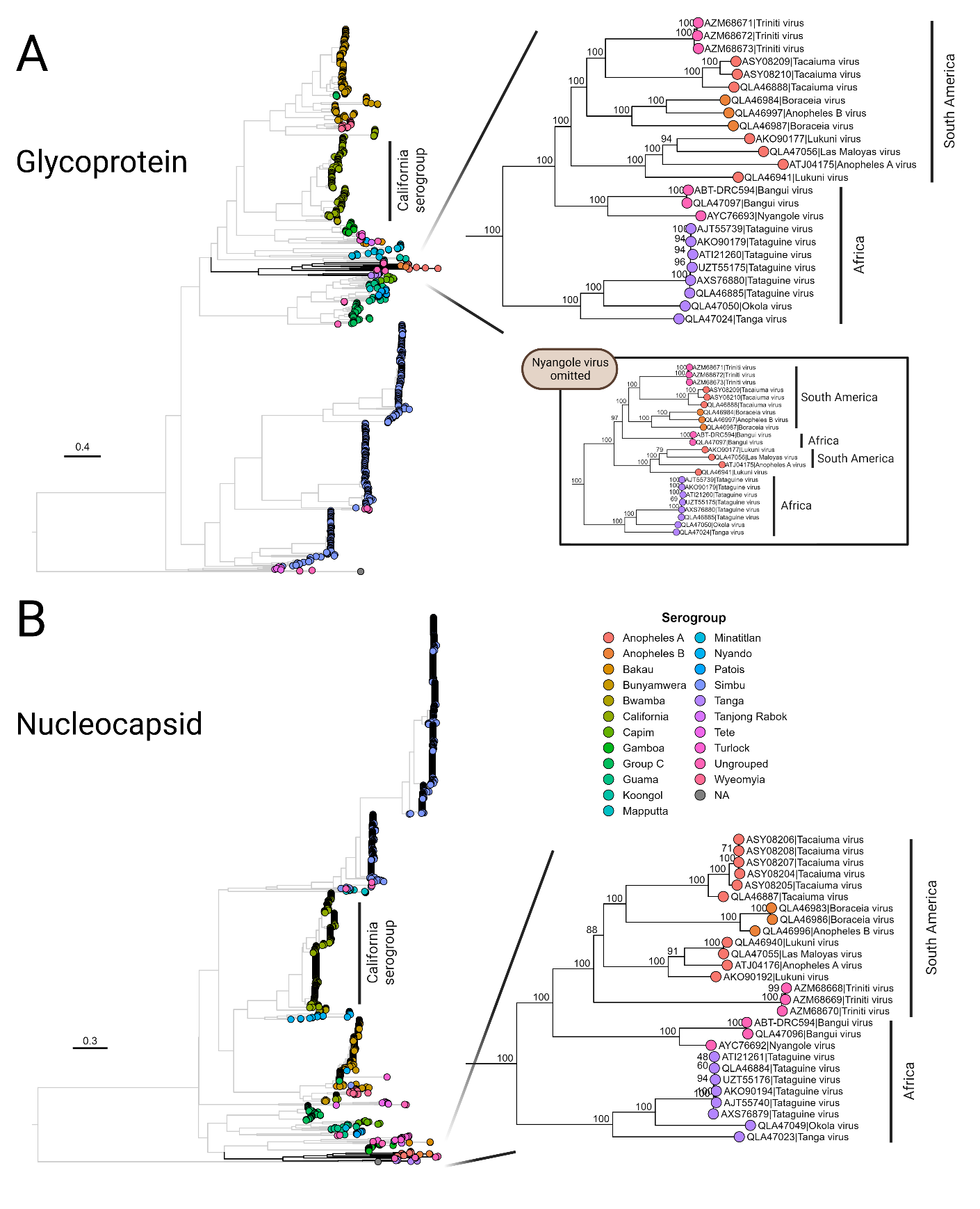


**Figure S1. ML reconstruction of the M and S segments of the Orthobunyavirus genus. A.** A total of 937 glycoprotein (M segment polyprotein containing the Gn, NSm, and Gc proteins) amino acid sequences are represented. **B.** A total of 1,435 nucleocapsid protein (S segment) amino acid sequences are represented. Each tree is rooted using Khurdun virus as an outgroup and the branch lengths are drawn to scale (measured in avg. substitutions/site). The tips are color-coded based on established or assumed serogroup. The monophyletic group containing the Tanga serogroup, Anopheles A/B serogroups, and Bangui-Nyangole clade is magnified on the right of each panel. In panel **A**, a separate calculation which omits Nyangole virus from the dataset is presented in the inset. Branch supports calculated using 1,000 replicates of ultrafast bootstrapping are shown at the nodes in the magnified portion of the tree.


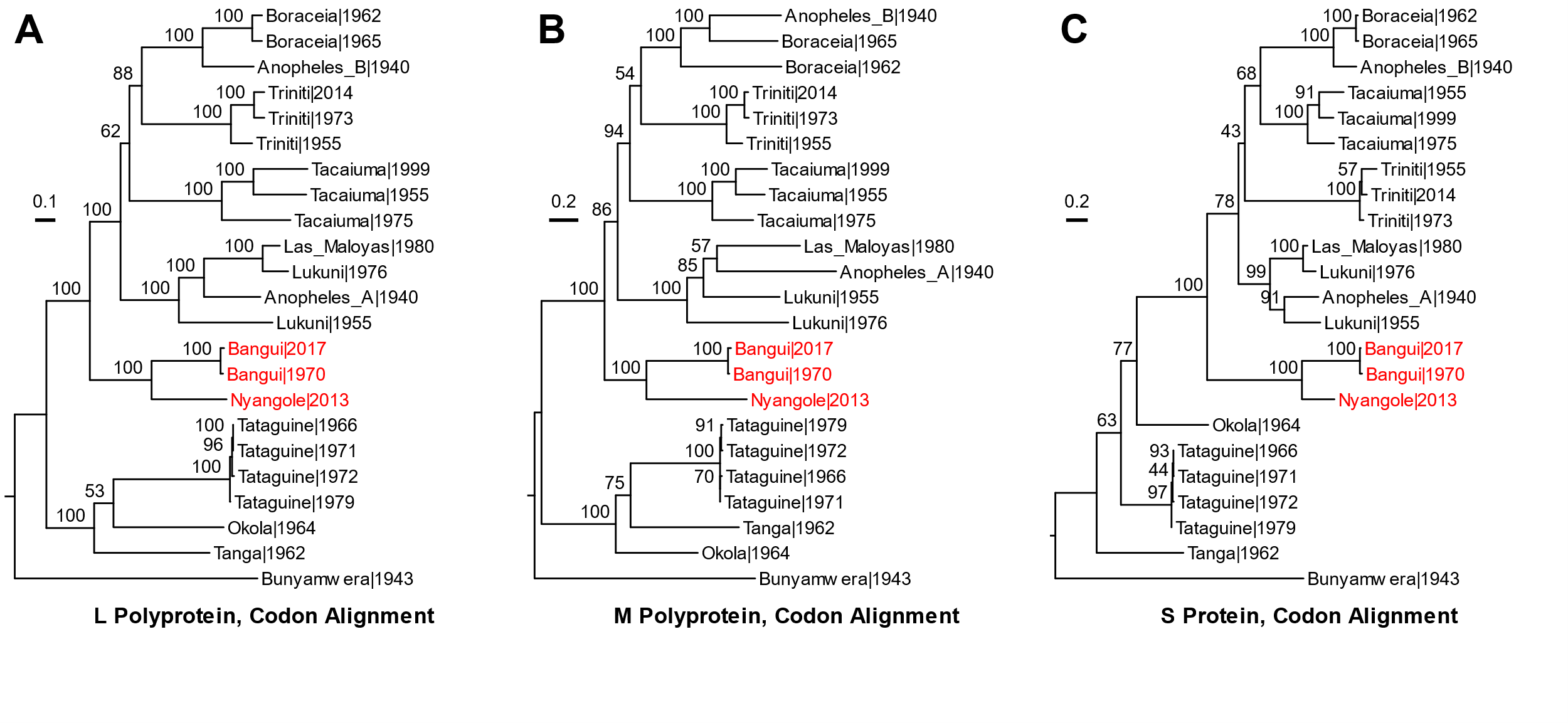
**Figure S2. ML reconstruction of the nucleotidic codon sequences encoding the major (poly)proteins of n=23 closely-related orthobunyaviral genomes. A.** The L polyprotein (RdRP), **B.** The M polyprotein (Gn, NSm, and Gc), **C.** The N protein. Each tree is rooted using Bunyamwera virus as an outgroup and the branch lengths are drawn to scale (measured in avg. substitutions/site). The Bangui-Nyangole clade is highlighted in red.


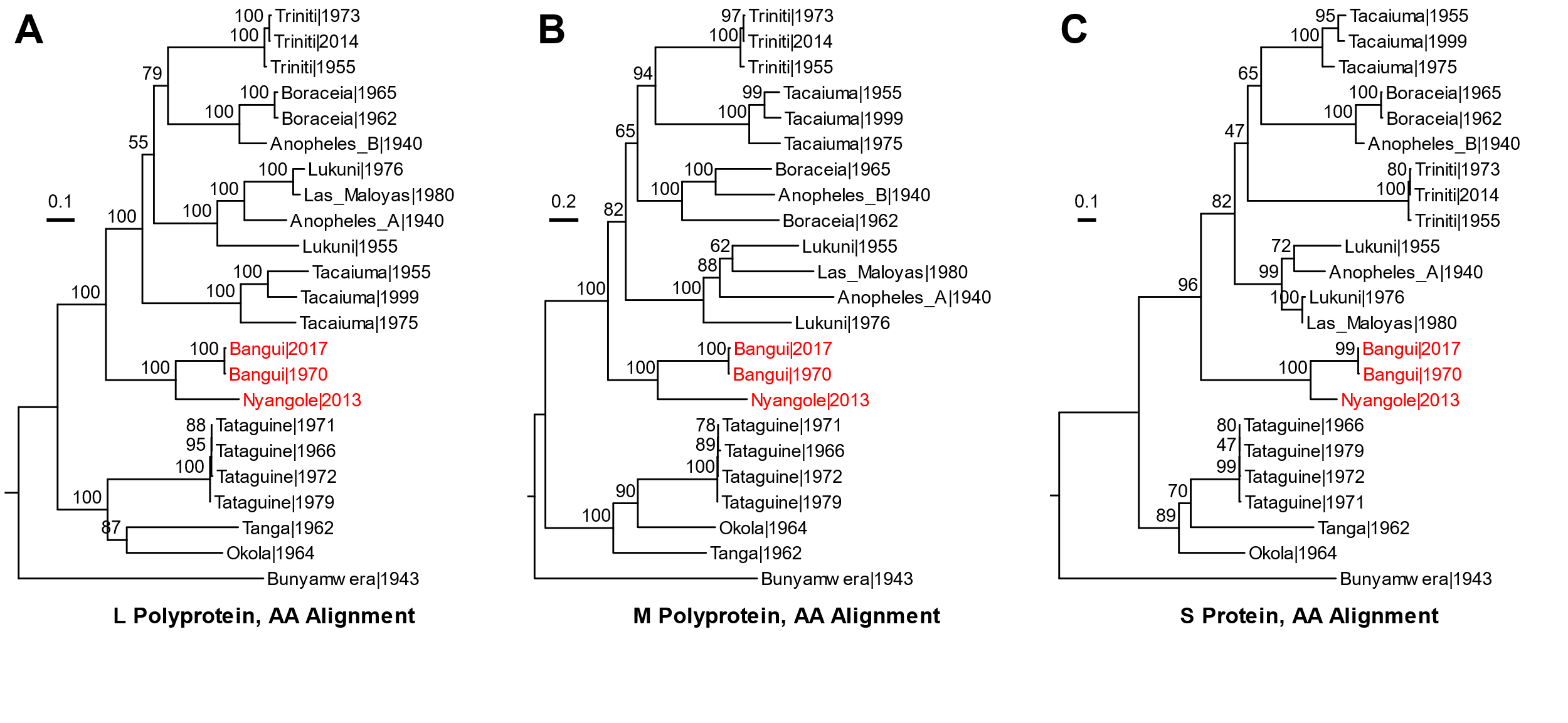
**Figure S3. ML reconstruction of the amino acid sequences of the major (poly)proteins of n=23 closely-related orthobunyaviral genomes. A.** The L polyprotein (RdRP), **B.** The M polyprotein (Gn, NSm, and Gc), **C.** The N protein. Each tree is rooted using Bunyamwera virus as an outgroup and the branch lengths are drawn to scale (measured in avg. substitutions/site). The Bangui-Nyangole clade is highlighted in red.


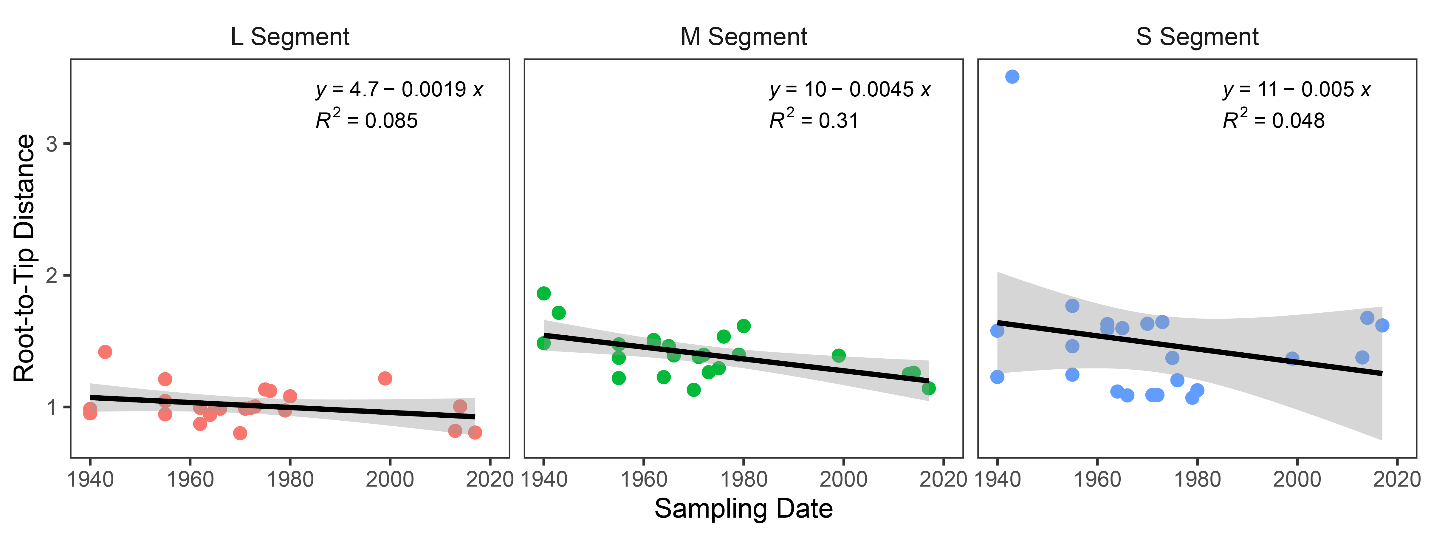


**Figure S4. Root-to-tip regression of maximum likelihood phylogenetic reconstructions of Dataset B.** The program TempEst was used to apply the heuristic residual mean squared to root each tree at the position most likely to be compatible with the assumption of a molecular clock. Linear regression of the relationship between the resulting root-to-tip distances and the virus collection dates was performed in R.

**Table S2. Reproduction of the Hughes et al. estimation of the evolutionary rates in California serogroup bunyaviruses.** Genbank accessions refer to nucleotide sequences for whole segments; the open reading frames for the L polyprotein, M polyprotein, and Nucleocapsid (N) protein were extracted from each, then aligned by MAFFT. Molecular dating of the nucleotide sequences of each segment, incorporating sampling times, was achieved using BEAST as described in the Materials and Methods section of the main text.

| **Segment** | **Accessions** | **Mean rate (substitutions/site/year)** |
| --- | --- | --- |
| **L** | KX817312, KX817315, HM007355, HM007358, KX817318, KX817321, KT630288, KT630291, KX817324, KX817327, KX817330, KX817333, KX817336, KF719235, EU616903, KY748357, AF528165, EF485032, EF485038, GU206146, GU591169, GU596376, EU203678, , HM243137, KF361878, HM036216, EU789573, KX554937, KR149249, KX891321 | 5.875E-05 |
| **M** | KX817313, KX817316, HM007354, HM007357, KX817319, KX817322, KT630289, KT630292, KX817325, KX817328, KX817331, KX817334, KX817337, JN815081, KF719234, EU621834, , AF528166, EF485031, EF485037, GU206145, GU591168, GU596381, EU262553, , HM243138, GQ386831, HM036215, U88059, KX554936, KR149248, KX891322 | 6.789E-05 |
| **S** | KX817314, U12800, KX817317, U12799, HM007353, HM007356, KX817320, KX817323, KT630290, KT630293, KX817326, KX817329, KX817332, KX817335, KX817338, GU018050, KF719233, EU479697, EU479698, GQ330486, AF528167, EF485030, EF485036, GU206144, GU591167, GU596386, EU294510, KM215563, KM215562, HM243139, GU390678, HM036214, U47137, KX554935, KR149247, KX891323 | 5.564E-05 |

**Table S3. Bayesian model selection using Nested Sampling (NS).** This evaluation considers two different priors and two different clocks for calibration of the Tanga/Anopheles group evolutionary rate using the established rates for the California serogroup. The log Bayes factor (BF) is computed as the difference between the log marginal likelihood estimation (MLE) obtained from using two different priors with the same clock or two different clocks using the same prior ($BF={MLE}_{1}-{MLE}_{2}$; if positive, MLE_1_ is favored). BF is considered significant (*) when greater than $(2\times\sqrt{{SD}_{1}^{2}+{SD}_{2}^{2}})$. SD: standard deviation. ^#^ denotes the prior was used with tip dating; the other priors were used without tip dating. The best prior and clock in each comparison are bolded.

| **Evaluation of Prior; Statistics** | | | | | |
| --- | --- | --- | --- | --- | --- |
| **Clock** | **Prior** | **NS Particle Count** | **Information** | **MLE** | **SD** |
| SC | BICEPS^#^ | 20 | 403.8537 | -163371.6206 | 4.4897 |
| SC | BICEPS | 20 | 379.5764 | -163347.4909 | 4.3564 |
| **SC** | **Yule Skyline** | **20** | **375.7131** | **-163345.3651** | **4.1738** |
|  |  |  |  |  |  |
| **Evaluation of Prior; Results** | | |  |  |  |
| **Comparison** | **Selected** | **BF** |  |  |  |
| BICEPS vs BICEPS^#^ | BICEPS | 24.1297* |  |  |  |
| Yule Skyline vs BICEPS^#^ | Yule Skyline | 26.2555* |  |  |  |
| Yule Skyline vs BICEPS | Yule Skyline | 2.1258 |  |  |  |
|  |  |  |  |  |  |
| **Evaluation of Clock; Statistics** | | | | | |
| **Clock** | **Prior** | **NS Particle Count** | **Information** | **MLE** | **SD** |
| SC | Yule Skyline | 20 | 375.7131 | -163345.3651 | 4.1738 |
| **URLC** | **Yule Skyline** | **20** | **447.4481** | **-163260.3757** | **4.7299** |
|  |  |  |  |  |  |
| **Evaluation of Clock; Results** | | |  |  |  |
| **Comparison** | **Selected** | **BF** |  |  |  |
| URLC vs SC | URLC | 84.9894* |  |  |  |
|  |  |  |  |  |  |
|  |  |  |  |  |  |

**Table S4. Pervasive positive selection evaluation estimated by site models in the CODEML program implemented in the PAML package.** Abbreviations: -lnL: negative log-marginal-likelihood

| **Segment** | **Model** | **-lnL** |  |  |  |  |  | **Positive site(s)** |
| --- | --- | --- | --- | --- | --- | --- | --- | --- |
| **L** | M0 | 85230.950906 | **Ω_0_ = 0.01262** |  |  |  |  | - |
|  | M1 | 84981.292161 | p0 = 0.96102 | p1 = 0.03898 | ω = 1.000 |  |  | - |
|  | M2 | 84981.292162 | **Ω_2_ = 1.000** | p0 = 0.96102 | p1 = 0.00211 | p2 = 0.03687 |  | - |
|  | M3 | 98973.773753 | **Ω_2_ = 0.05234** | p0 = 0.97647 | p1 = 0.00120 | p2 = 0.02233 |  | - |
|  | M7 | 81863.034690 | p = 0.52187 | q = 34.54522 |  |  |  | - |
|  | M8 | 81852.697994 | **Ω_2_ = 1.0000** | p0 = 0.99911 | p = 0.52076 | q = 34.72181 | (p1 = 0.00089) | - |
| **M** | M0 | 67669.825275 | **Ω_0_ = 0.02329** |  |  |  |  |  |
|  | M1 | 67472.178655 | p0 = 0.93613 | p1 = 0.06387 |  |  |  | - |
|  | M2 | 67472.178655 | **Ω_2_ = 1.000** | p0 = 0.93613 | p1 = 0.00012 | p2 = 0.06375 |  | - |
|  | M3 | 65662.887689 | **Ω_2_ = 0.01472** | p0 = 0.20717 | p1 = 0.41871 | p2 = 0.37413 |  | - |
|  | M7 | 65640.875721 | p = 0.87666 | q = 37.48595 |  |  |  | - |
|  | M8 | 65639.074773 | **Ω_2_ = 1.0000** | p0 = 0.99835 | p = 0.88258 | q = 39.35845 | (p1 = 0.00165) | - |
| **S** | M0 | 9167.750874 | **Ω_0_ = 0.01081** |  |  |  |  |  |
|  | M1 | 9162.709072 | p0 = 0.99178 | p1 = 0.00822 |  |  |  | - |
|  | M2 | 9162.709070 | **Ω_2_ = 1.000** | p0 = 0.99178 | p1 = 0.00439 | p2 = 0.00383 |  | - |
|  | M3 | 8896.402415 | **Ω_2_ = 0.59790** | p0 = 0.29520 | p1 = 0.23228 | p2 = 0.47252 |  | - |
|  | M7 | 8893.617752 | p = 0.74956 | q = 61.13766 |  |  |  | - |
|  | M8 | 8886.003378 | **Ω_2_ = 2.14985** | p0 = 0.99999 | p = 0.88258 | q = 39.35845 | (p1 = 0.00165) | - |

**Table S5. Episodic positive selection evaluation estimated by the branch-site models of the CODEML program implemented in the PAML software package.** Shaded columns represent those clades which show statistically-significant evidence of positive selection when selected as foreground branches. Abbreviations: -lnL: negative log-likelihood; n.m.: null model; a.m.: alternative model.

| **Segment** | **Clade** | **Ω_2_** | **-lnL^a.m.^** | **-lnL^n.m.^** | **\|2ΔlnL\|** |
| --- | --- | --- | --- | --- | --- |
| **L** | I | 1.00000 | 84818.87002 | 84818.87004 | 0.00004 |
|  | II | 1.00000 | 84842.73314 | 84842.73308 | 0.00012 |
|  | **III** | **4.50832** | **85700.52978** | **84916.91715** | **1567.22526** |
|  | IV | 1.00000 | 84819.20070 | 84819.20066 | 0.00008 |
|  | V | 1.00000 | 84845.08461 | 84845.08458 | 0.00006 |
|  | VI | 1.00000 | 84802.60544 | 84802.60544 | 0.00000 |
|  | VII | 1.00000 | 85122.82196 | 85122.82193 | 0.00006 |
|  | VIII | 1.00000 | 84994.03454 | 84994.03454 | 0.00000 |
|  | **IX** | **2.94375** | **108833.38698** | **84797.65118** | **48071.47160** |
|  | **X** | **4.52492** | **100918.10129** | **84998.21695** | **31839.76868** |
|  | XI | 1.00000 | 84981.23310 | 84999.10325 | 35.74030 |
|  | XII | 1.00000 | 85052.52188 | 85077.39434 | 49.74492 |
|  | XIII | 1.00000 | 85022.91012 | 85054.20479 | 62.58934 |
| **M** | I | 1.00000 | 67412.95359 | 67412.95359 | 0.00000 |
|  | **II** | **25.42423** | **67316.58499** | **67333.60681** | 34.04364 |
|  | III | 1.00000 | 67421.22059 | 67410.80666 | 20.82786 |
|  | IV | 1.00000 | 67411.35543 | 67411.35543 | 0.00000 |
|  | V | 1.00000 | 67362.12946 | 67362.12946 | 0.00000 |
|  | VI | 1.00000 | 67430.83094 | 67529.00255 | 196.34322 |
|  | VII | 1.00000 | 67260.66345 | 67108.37330 | 304.58030 |
|  | VIII | 1.00000 | 67624.01071 | 67500.79857 | 246.42428 |
|  | **IX** | **90.15419** | **67636.00453** | **67443.01926** | **385.97054** |
|  | X | 1.00000 | 67611.70933 | 67463.82697 | 295.76472 |
|  | XI | 1.00000 | 67642.94195 | 67476.88452 | 332.11486 |
|  | **XII** | **23.43435** | **67475.55572** | **67292.27016** | **366.57112** |
|  | XIII | 1.00000 | 67683.69387 | 67443.01927 | 481.34920 |
| **S** | I | 1.00000 | 9127.41153 | 9127.405267 | 0.01253 |
|  | II | 7.80676 | 9138.97228 | 9140.64036 | 3.33616 |
|  | III | 1.00000 | 9134.94805 | 9142.49083 | 15.08556 |
|  | IV | 7.59156 | 9148.79088 | 9148.79715 | 0.01254 |
|  | V | 1.00000 | 9133.74264 | 9133.74890 | 0.01252 |
|  | VI | 1.00000 | 9153.52939 | 9155.02549 | 2.99220 |
|  | VII | 1.00000 | 9168.10950 | 9158.78773 | 18.64354 |
|  | **VIII** | **53.35526** | **9157.71069** | **9155.57403** | **4.27332** |
|  | IX | 1.00000 | 9160.64377 | 9162.17320 | 3.05886 |
|  | X | 1.00000 | 9136.96643 | 9142.52871 | 11.12456 |
|  | XI | 1.00000 | 9161.72591 | 9162.35449 | 1.25716 |
|  | XII | 1.00000 | 9168.10950 | 9168.11579 | 0.01258 |
|  | XIII | 1.00000 | 9168.10960 | 9168.11577 | 0.01234 |
